# ASO Author Reflections: Implementation of Age and Co-morbidity in the Treatment Guideline of Patients with Esophageal Squamous Cell Carcinoma

**DOI:** 10.1245/s10434-019-07361-4

**Published:** 2019-04-22

**Authors:** Z. Faiz, J. T. M. Plukker

**Affiliations:** Department of Surgery/Surgical Oncology, University of Groningen, University Medical Center Groningen, Groningen, The Netherlands

## Past

Esophagectomy following neoadjuvant chemoradiotherapy (nCRT) remains standard treatment for patients with potentially curable locally advanced esophageal cancer (EC). In the CROSS study with carboplatin/paclitaxel and 41.4 Gy/23 × 1.8 Gy, a pathologic complete response was achieved in 23% and 49% of patients with esophageal adenocarcinoma (EAC) and squamous cell carcinoma (ESCC), respectively.^1^

However, high-aged patients and those with severe comorbidity are faced with considerable high postoperative morbidity and mortality.^2^ In these patients, who are medically unfit for surgery, definitive chemoradiotherapy (dCRT) would be a good alternative curative-intended treatment.^3^ Most studies in the past explored the usefulness of cisplatin-based regimen according to the RTOG 85-01 landmark study. Recent studies have shown more or less equal results of carboplatin/paclitaxel-based dCRT. In contrast with ESCC, data concerning the efficacy of dCRT in EAC are still lacking. Besides some recommendation, age and comorbidity are not clearly implemented in current treatment guideline of patients with EC.^4^,^5^

## Present

Many elderly patients have multiple age-associated comorbidities, limiting the use of current combined treatment with either nCRT or dCRT. In our study age ≥ 75 years and multiple comorbidities were associated with a higher probability for dCRT. Approximately 78% of these elderly patients were treated with dCRT.^6^ The strongest associations were found for the combination of hypertension plus diabetes and the combination of cardiovascular with pulmonary comorbidity. The results of this population-based study support the administration of dCRT in patients with ESCC having at least two comorbidities or being older than 75 years. This was seen particularly among those with cardiovascular diseases or previous malignancies, because their overall survival after dCRT was comparable to the overall survival for patients after nCRT plus surgery. However, in operable patients with locally advanced EAC, the use of nCRT plus surgery was associated with a better overall survival regardless of age, number, and type of pretreatment comorbidities.

## Future

In a selected group of elderly patients following dCRT, good results are reported with complete responses (58–68%) and 2-year survival rates of 36–64% against acceptable ≥ grade 3 toxicity (24–36%).^7^ Several studies have stressed better results with dCRT in ESCC and the use of carboplatin/paclitaxel regimen with less toxicity and similar results compared with cisplatin-based dCRT.^8^,^9^

As functional rather than chronological older age is decisive for a proper treatment decision-making, comprehensive geriatric assessment is required in multidisciplinary tumor boards. Moreover, the increased risk of postoperative treatment-related morbidity and mortality in these patients is associated with the frailty index. Although there is no consensus on the definition of frailty and standardized cutoff points, comprehensive frailty testing facilitates an individualized preoperative risk assessment, while improving clinical outcome.^10^

Promising strategies are the use of biomarkers in combined chemoimmunotherapy as (neo)adjuvant,^11^ whereas improved outcome and less toxicity might be achieved by up-to-date radiation techniques, including intensity-modulated radiotherapy and proton therapy.^12^
